# The Society of Homœopathicians

**Published:** 1894-11

**Authors:** 


### THE SOCIETY OF HOMŒOPATHICIANS.

### A NEW SOCIETY.

"*I have no use for Homœopathy except the name.*"—PRETENDER.

"*Who would honor such a light-minded and pernicious sect by calling them, after the difficult yet beneficent art, homœopathic physician?*" (Hahnemann's *Organon*, foot-note, Sec. 149.)

In the year 1790 Hahnemann discovered and promulgated the sufficient and unchangeable natural law of cure provided for the relief of the sick, and he named the practice based upon this law Homœopathy.

From that time down to the present a large class of men who, while pluming themselves with the name, have, either through blind or willful ignorance, persistently evaded the application of the law in practice, and many of this class, with a foolish desire to appear to be "abreast of the times" with the enemies of Homœopathy, whose ridicule they incur, vainly profess to believe that they in their wisdom can supersede this law in practice. History, therefore, relates the fact of the subversion of all the societies or organizations that have been instituted for the advancement of Homœopathy. Not only have these organizations been subverted, but, as is to be expected in these days, a nnmber have been established by the influence of the evil one to further a deception.

Two instances may be named as sufficient to demonstrate the experience in the life and ultimate result of the associations that have been formed for the advancement of Homœopathy. They are the first and the last, namely : The American Institute of Homœopathy and the International Hahnemannian Association. Both were organized by men whose names will endure forever, and yet, notwithstanding their teaching and their influence, these organizations have been driven into chaotic confusion by the inroad of the worst enemies of Homœopathy, the pretenders.

At the meeting held at Narragansett Pier, June 21st, 1892, the I. H. A. deliberately violated its Declaration of Principles, Constitution, and By-Laws by voting to indefinitely postpone a report of the Board of Censors before it was submitted, in order to shield a member in error.

This repudiation of its principles destroyed the life of pure Homœopathy as represented by the I. H. A., and no "resolution" can restore that life or the integrity of the Association.

A new society is, therefore, imperative for the preservation and advancement of pure Homœopathy, in which adherence to principles will be regarded of more importance than personal feelings.

This society gives promise of success in avoiding the mistakes in the organination of former societies, which experience has taught to be chiefly due to a reckless admission of members. The following Declaration of Principles, Constitution, and By-Laws, it is believed, gives this assurance.

### Declaration of Principles.

I. Disease being a disturbance of the spirit-like life force, is manifested by symptoms subjective and objective.

II. The healing power of medicines is ascertained by proving them upon the healthy and by clinical experience.

III. Disease is most effectively, safely, and rapidly cured by potentiated remedies corresponding with the spirit-like life force.

IV. The curative relation of the medicine to the disturbed life force depends upon the similarity of symptoms and upon the potency.

V. The formula *Similia Similibus Curantur* is the empirical proposition of the fact that like cures like. It is verified by every cured case and is the guide to the selection of the remedy. Being co-extensive with the entire realm of healing by remedial forces, it serves as the law of cure.

VI. The law of cure is the application in medicine of the principle of mutual action contained in the Newtonian law of motion: Action and reaction are equal and contrary; or the actions of two bodies are in themselves mutually equal and directed to contrary sides. (*Phil. Nat. Principia Math.* London, 1687, p. 12.)

### Rules for Practice.

I. Only one remedy at a time is to be given.

II. Remedies are best given in potentiated form.

III. Surgical treatment is indicated only as stated by Hahnemann in Section 186 of *The Organon.*

IV. Suppression of symptoms by crude medicines, local treatment, or by any other means is unhomœopathic.

### Constitution.

Article I. This Society shall be known as the Society of Homœopathicians.

Art. II. Its objects shall be the study and dissemination of the principles of Homœopathy, as promulgated by Samuel Hahnemann, and the study of general medicine.

Art. III. The officers shall consist of an Executive Board of five members, a Secretary, and Treasurer.

Art. IV. Applicants for membership shall be graduates of a recognized medical college; shall be of good moral character; shall have been in the active practice of Homœopathy for three years, and shall indorse the Declaration of Principles and Rules for Practice adopted by this Society.

### By-Laws.

Section I. This Society shall meet annually at such time and place as may be determined by the Executive Board.

Sec. II. The Secretary and Treasurer shall be elected annually by ballot; their duties shall be the same as in similar associations. The Secretary shall also act as secretary of the Executive Board.

Sec. III. 1. The Executive Board shall be elected in the first instance, one for one year, one for two years, one for three years, one for four years, and one for five years. At each annual meeting thereafter one member shall be elected by ballot for the full term of five years.

2. The Executive Board shall have charge of all business not otherwise provided for, shall hold in trust all property, papers for publication, shall appoint the chairman of the various Bureaus, and shall perform such other duties as may be assigned them by vote of the Association. They shall elect their own chairman, fill vacancies in their own body, and shall also act as the Board of Censors.

3. Any paper adjudged worthy of publication by the Executive Board shall be published as written by the author. All corrections and alterations of papers must be made by the authors for submission to the Board. The author may require printer's proof before publication.

4. A quorum of the Executive Board shall consist of three members, and any member who shall be absent from two consecutive meetings of the Society, except by reason of sickness or absence from home, shall no longer be a member of the Board, and the Board may elect to fill the vacancy.

Sec. IV. At each annual meeting a Chairman shall be elected to preside at the sessions of that meeting.

He shall appoint all committees not otherwise ordered.

Sec. V. Applications for membership may be received at any time and shall be indorsed by three members in good standing.

Said indorsement must be made not upon the general reputation of the applicant, but *from positive knowledge* of at least one of the indorsers as to the integrity of his homœopathic practice.

SEC. VI. The application shall be in the possession of the Chairman of the Executive Board at least six months before the next annual meeting, and the applicant shall send to the said chairman, three months before the next annual meeting, a clinical report of three cases treated by said applicant, and shall furnish satisfactory evidence that he keeps a clinical record of his cases.

SEC. VII. The Executive Board shall send the names of all the applicants, with their indorsers, to each member of the Society at least three months before the next annual meeting.

SEC. VIII. If the application is favorably considered by the Executive Board, the Chairman of the Board shall notify the said applicant of the time and place of the next annual meeting, at least one month before, and shall invite him to personally read an original paper before the meeting upon such subject as he may choose.

SEC. IX. At the next annual meeting after the one at which the applicant read his paper, and upon the unanimous recommendation of the Executive Board, an election by ballot shall be held and three negative ballots shall be necessary to reject the applicant. If an applicant be rejected, said applicant may make a second application at the expiration of two years.

SEC. X. The number of applicants to be recommended by the Executive Board for election to membership shall be limited to five yearly.

SEC. XI. The number of members of the Society shall be limited to fifty.

SEC. XII. The annual dues shall be five dollars, payable in advance. Any member who shall fail to pay the annual dues shall, for the time they remain unpaid, forfeit all privileges of membership except by unanimous consent of the members present.

All members whose dues shall remain unpaid for more than two years without giving satisfactory reasons therefor shall be dropped from the roll of membership until such dues are paid in full.

SEC. XIII. The Executive Board shall investigate charges in writing preferred against any member for un-homœopathic practice, or for advocating practices contrary to the Declaration of Principles and Rules for Practice of this Society, and shall report to the Association the results of such investigation for final action.

The Society, by a two-thirds' vote of the members, present shall have power to expel.

SEC. XIV. The Constitution and By-Laws may be amended at any annual meeting of this Society by a two-thirds' vote of the members present, notice having been given in writing at a previous annual meeting.

The following is a list of the members and officers of the Society: Edward Adams, M. D.; J. A. Biegler, M. D.; Edmund Carleton, M. D.; Stuart Close, M. D.; O. M. Drake, M. D.; F. S. Davis, M. D.; B. Fincke, M. D.; S. Mills Fowler, M. D.; J. R. Haynes, M. D.; A. L. Kennedy, M. D.; S. A. Kimball, M. D.; F. W. Patch, M. D.; F. O. Pease, M. D.; E. E. Reininger, M. D.; E. W. Sawyer, M. D.; J. W. Thomson, M. D.; J. A. Tomhagen, M. D.; R. L. Thurston, M. D.

The Executive Board consists of: J. A. Biegler, M. D.; Edmund Carleton, M. D.; S. A. Kimball, M. D.; E. W. Sawyer, M. D.; R. L. Thurston, M. D. Secretary, S. A. Kimball, M. D.; Treasurer, F. S. Davis, M. D.

